# Supplementary material for: Oral-Health-Related Quality of Life in Patients with Medication-Related Osteonecrosis of the Jaw: A Prospective Clinical Study
Source: Int J Environ Res Public Health. 2022 Sep 16;19(18):11709. doi: 10.3390/ijerph191811709 (PMC9517310; doi:10.3390/ijerph191811709)
Supplement: Supplementary file 1 [file ijerph-19-11709-s001.zip › Table S1.pdf]

Table S1. Descriptive data of the OHIP domains functional limitation and psychological discomfort from the seven-domain OHIP structure. SD: Standard deviation.

OHIP Domain		Functional Limitation						Psychological Discomfort					
Time of Assessment		T0		T1		T2		T0		T1		T2	
Parameter	Groups	Mean	SD	Mean	SD	Mean	SD	Mean	SD	Mean	SD	Mean	SD
Total	-	13.0	6.9	11.0	5.6	9.8	6.3	9.9	5.4	6.4	4.1	5.6	4.0
Stage	I	13.8	6.9	11.2	5.8	10.5	6.7	10.3	5.1	6.4	3.9	6.1	4.2
	II	11.3	6.9	10.5	5.6	8.3	5.0	8.9	6.1	6.5	4.6	4.6	3.3
Pain	no	12.4	5.0	10.3	5.9	9.1	6.0	8.6	4.3	6.4	4.1	5.5	3.3
	yes	13.4	8.1	11.5	5.5	10.3	6.6	10.8	5.9	6.4	4.1	5.7	4.5
Primary disease	osteoporosis	15.9	5.5	10.5	3.8	9.4	5.0	12.3	5.1	6.0	3.5	5.3	3.7
	malignoma	12.2	7.2	11.1	6.1	10.0	6.7	9.2	5.3	6.5	4.2	5.7	4.1
Risk evaluation	low risk	14.9	4.8	9.8	3.8	8.3	4.9	12.1	4.3	5.8	3.4	5.5	3.8
	high risk	12.0	7.7	11.6	6.4	10.5	6.8	8.8	5.6	6.7	4.4	5.7	4.1
Duration of intake	short	11.6	5.3	11.5	5.4	10.3	4.2	8.5	4.5	6.9	4.3	6.5	2.9
	long	14.1	7.8	10.5	5.9	9.4	7.5	10.8	5.8	6.1	3.9	5.0	4.6
Defect size	small	12.3	6.0	11.2	2.2	10.1	3.9	10.6	4.1	8.0	3.3	6.8	3.3
	medium	12.8	8.0	12.4	8.1	10.5	9.2	9.6	5.3	6.1	4.5	4.9	5.0
	large	13.7	7.2	9.7	5.3	9.0	5.3	9.5	6.5	5.4	4.2	5.2	3.6
Need for prosthodontics	yes	15.9	7.3	12.7	6.3	11.4	7.1	11.8	4.9	7.5	4.2	6.7	4.3
	no	10.4	5.6	9.5	4.6	8.4	5.2	8.2	5.3	5.4	3.8	4.6	3.4
