# Supplementary material for: Oral-Health-Related Quality of Life in Patients with Medication-Related Osteonecrosis of the Jaw: A Prospective Clinical Study
Source: Int J Environ Res Public Health. 2022 Sep 16;19(18):11709. doi: 10.3390/ijerph191811709 (PMC9517310; doi:10.3390/ijerph191811709)
Supplement: Supplementary file 1 [file ijerph-19-11709-s001.zip › Table S2.pdf]

Table S2. Descriptive data of the OHIP domains physical pain and physical disability from the seven-domain OHIP structure. SD: Standard deviation.

OHIP Domain		Physical Pain						Physical Disability					
Time of Assessment		T0		T1		T2		T0		T1		T2	
Parameter	Groups	Mean	SD	Mean	SD	Mean	SD	Mean	SD	Mean	SD	Mean	SD
Total	-	16.1	8.1	10.9	6.2	9.3	6.0	11.4	8.1	10.3	7.0	9.1	5.9
Stage	I	15.4	7.5	10.7	6.1	9.7	6.4	12.1	7.8	10.8	6.7	10.0	6.1
	II	17.7	9.4	11.5	6.6	8.4	5.3	10.0	9.1	9.1	7.9	6.9	5.0
Pain	no	14.9	8.3	9.5	4.2	8.3	5.8	10.3	6.3	10.0	7.2	8.9	5.1
	yes	17.0	8.0	11.9	7.2	10.0	6.2	12.2	9.3	10.5	7.1	9.1	6.5
Primary disease	osteoporosis	17.8	4.3	10.0	4.7	8.3	4.5	14.9	6.7	11.0	4.0	10.0	7.1
	malignoma	15.6	8.9	11.2	6.6	9.6	6.4	10.5	8.4	10.1	7.7	8.8	5.6
Risk evaluation	low risk	20.5	6.3	10.7	4.4	9.0	6.8	14.2	6.8	9.3	4.3	8.8	6.7
	high risk	13.9	8.1	11.0	7.0	9.4	5.8	10.1	8.5	10.8	8.1	9.2	5.6
Duration of intake	short	15.7	8.8	10.7	6.0	10.0	5.7	8.5	6.8	10.3	7.9	9.8	3.8
	long	16.3	7.8	11.1	6.4	8.8	6.3	13.5	8.5	10.2	6.5	8.5	7.1
Defect size	small	15.7	6.9	12.5	5.3	11.1	7.4	11.1	7.6	11.5	5.4	10.4	3.7
	medium	16.1	7.9	11.1	7.6	7.9	6.2	12.9	9.6	11.6	9.3	8.6	6.7
	large	16.4	9.5	9.6	5.8	8.9	4.7	10.6	7.7	8.3	6.0	8.4	6.8
Need for prosthodontics	yes	19.8	8.3	12.4	7.1	10.2	7.2	14.3	8.4	12.6	7.8	10.2	7.1
	no	12.8	6.5	9.6	5.2	8.5	4.8	8.9	7.2	8.2	5.7	8.1	4.5
