# Supplementary material for: Oral-Health-Related Quality of Life in Patients with Medication-Related Osteonecrosis of the Jaw: A Prospective Clinical Study
Source: Int J Environ Res Public Health. 2022 Sep 16;19(18):11709. doi: 10.3390/ijerph191811709 (PMC9517310; doi:10.3390/ijerph191811709)
Supplement: Supplementary file 1 [file ijerph-19-11709-s001.zip › Table S3.pdf]

Table S3. Descriptive data of the OHIP domains psychological disability and social disability from the seven-domain OHIP structure. SD: Standard deviation.

OHIP Domain		Psychological Disability						Social Disability					
Time of Assessment		T0		T1		T2		T0		T1		T2	
**Parameter**	**Groups**	**Mean**	**SD**	**Mean**	**SD**	**Mean**	**SD**	**Mean**	**SD**	**Mean**	**SD**	**Mean**	**SD**
Total	-	7.6	5.6	5.4	3.9	5.6	4.5	5.0	5.0	3.7	3.8	4.3	4.3
Stage	I	8.2	5.5	6.1	3.9	6.2	4.9	5.4	5.4	3.8	4.1	4.4	4.5
	II	6.5	5.8	3.8	3.7	4.3	3.3	3.9	4.1	3.5	3.4	3.8	3.7
Pain	no	6.1	4.2	4.9	3.2	4.4	3.2	4.3	3.8	2.8	3.1	3.4	3.3
	yes	8.7	6.3	5.8	4.4	6.4	5.2	5.5	5.8	4.3	4.2	4.9	4.8
Primary disease	osteoporosis	11.5	4.9	6.9	3.3	6.1	4.5	7.6	5.2	4.9	4.7	4.1	4.9
	malignoma	6.5	5.4	5.0	4.1	5.4	4.6	4.2	4.8	3.4	3.6	4.3	4.1
Risk evaluation	low risk	10.4	5.0	5.7	3.3	5.7	4.1	6.1	4.9	3.8	4.3	3.5	4.3
	high risk	6.3	5.5	5.3	4.3	5.5	4.8	4.4	5.1	3.7	3.7	4.6	4.3
Duration of intake	short	5.3	4.0	4.4	3.3	5.0	2.3	3.6	4.1	3.1	3.3	4.0	3.1
	long	9.3	6.1	6.1	4.3	6.0	5.6	6.0	5.5	4.1	4.2	4.4	4.9
Defect size	small	8.6	5.8	6.6	3.2	6.7	3.6	6.3	6.1	4.6	4.1	4.6	4.3
	medium	7.6	5.5	6.0	4.5	5.3	6.6	5.1	5.4	4.2	4.1	4.6	5.6
	large	6.9	5.8	3.9	3.8	4.9	3.5	3.9	3.9	2.6	3.5	3.8	3.2
Need for prosthodontics	yes	8.9	5.8	6.2	4.1	6.6	5.2	5.5	4.7	4.1	4.0	4.7	4.5
	no	6.5	5.4	4.7	3.7	4.7	3.7	4.5	5.4	3.4	3.8	3.9	3.8
