# Supplementary material for: Oral-Health-Related Quality of Life in Patients with Medication-Related Osteonecrosis of the Jaw: A Prospective Clinical Study
Source: Int J Environ Res Public Health. 2022 Sep 16;19(18):11709. doi: 10.3390/ijerph191811709 (PMC9517310; doi:10.3390/ijerph191811709)
Supplement: Supplementary file 1 [file ijerph-19-11709-s001.zip › Table S4.pdf]

Table S4. Descriptive data of the OHIP domain handicap from the seven-domain OHIP structure. SD: Standard deviation.

**OHIP Domain**		**Handicap**					
**Time of Assessment**		**T0**		**T1**		**T2**	
**Parameter**	**Groups**	**Mean**	**SD**	**Mean**	**SD**	**Mean**	**SD**
Total	-	7.1	5.3	6.0	5.0	5.8	4.8
Stage	I	7.7	5.4	6.4	5.1	6.2	5.0
	II	5.7	4.8	5.2	4.7	4.7	4.2
Pain	no	6.2	4.2	5.9	4.9	5.1	4.6
	yes	7.8	5.9	6.1	5.1	6.2	5.0
Primary disease	osteoporosis	9.5	5.2	7.5	4.5	5.9	5.4
	malignoma	6.4	5.2	5.6	5.1	5.7	4.7
Risk evaluation	low risk	8.3	4.9	5.8	4.6	6.0	5.4
	high risk	6.5	5.5	6.1	5.2	5.6	4.6
Duration of intake	short	4.8	3.9	5.3	4.9	6.1	3.6
	long	8.8	5.6	6.5	5.1	5.5	5.5
Defect size	small	7.7	5.8	7.4	4.4	8.2	4.9
	medium	6.8	5.7	6.6	5.7	5.2	5.4
	large	6.9	4.9	4.4	4.7	4.3	3.7
Need for prosthodontics	yes	8.1	5.1	7.3	5.3	6.6	5.1
	no	6.2	5.4	4.8	4.5	5.0	4.5
