# Supplementary material for: Oral-Health-Related Quality of Life in Patients with Medication-Related Osteonecrosis of the Jaw: A Prospective Clinical Study
Source: Int J Environ Res Public Health. 2022 Sep 16;19(18):11709. doi: 10.3390/ijerph191811709 (PMC9517310; doi:10.3390/ijerph191811709)
Supplement: Supplementary file 1 [file ijerph-19-11709-s001.zip › Table S5.pdf]

Table S5. Descriptive data from the OHIP dimensions oral function and orofacial pain from the four dimensional OHIP scale. SD: Standard deviation.

**OHIP Dimension**		**Oral Function**						**Orofacial Pain**					
**Time of Assessment**		**T0**		**T1**		**T2**		**T0**		**T1**		**T2**	
**Parameter**	**Groups**	**Mean**	**SD**	**Mean**	**SD**	**Mean**	**SD**	**Mean**	**SD**	**Mean**	**SD**	**Mean**	**SD**
Total	-	13.3	8.9	12.5	8.4	10.2	7.8	13.1	6.7	8.2	4.9	6.9	5.3
Stage	I	12.5	8.8	11.1	7.6	8.6	7.1	13.1	5.8	8.1	4.0	5.8	4.9
	II	14.9	9.4	15.7	9.3	14.0	8.1	13.4	8.8	8.3	6.7	9.6	5.4
Pain	no	12.9	7.7	10.4	6.6	10.2	7.3	14.1	6.1	7.9	4.6	8.3	6.1
	yes	13.5	9.9	14.0	9.4	10.2	8.3	12.6	7.2	8.4	5.2	5.9	4.5
Primary disease	osteoporosis	14.6	11.2	12.3	9.3	9.6	9.1	11.1	6.3	5.8	4.2	5.1	4.6
	malignoma	12.9	8.4	12.6	8.3	10.4	7.5	13.8	6.8	8.6	4.9	7.5	5.4
Risk evaluation	low risk	16.6	11.8	14.7	9.8	12.5	10.0	12.5	6.6	7.5	6.5	6.8	5.2
	high risk	11.6	6.7	11.9	7.8	9.1	6.3	13.5	6.9	8.5	4.0	7.0	5.4
Duration of intake	short	15.0	6.1	13.9	8.1	10.7	6.9	12.1	6.5	7.9	4.9	6.9	4.6
	long	12.0	10.4	11.6	8.7	9.9	8.5	14.0	6.7	11.6	8.7	9.9	8.5
Defect size	small	14.0	10.0	12.5	8.6	10.9	7.4	11.8	4.6	8.5	3.8	6.6	3.1
	medium	12.7	8.4	9.6	9.7	7.7	7.5	12.4	7.0	7.7	4.5	7.5	5.8
	large	13.1	9.1	14.8	7.0	11.6	8.3	14.9	7.9	8.3	6.1	6.7	6.5
Need for prosthodontics	yes	15.6	9.9	14.4	9.0	12.8	8.4	13.4	6.7	7.8	5.6	6.8	6.2
	no	11.2	7.6	10.8	7.7	7.9	6.5	13.1	6.9	8.5	4.2	7.1	4.5
