# Supplementary material for: Oral-Health-Related Quality of Life in Patients with Medication-Related Osteonecrosis of the Jaw: A Prospective Clinical Study
Source: Int J Environ Res Public Health. 2022 Sep 16;19(18):11709. doi: 10.3390/ijerph191811709 (PMC9517310; doi:10.3390/ijerph191811709)
Supplement: Supplementary file 1 [file ijerph-19-11709-s001.zip › Table S6.pdf]

Table S6. Descriptive data from the OHIP dimensions orofacial appearance and psychosocial impact from the four dimensional OHIP scale. SD: Standard deviation.

OHIP Dimension		Orofacial Appearance						Psychosocial Impact					
**Time of Assessment**		**T0**		**T1**		**T2**		**T0**		**T1**		**T2**	
**Parameter**	**Groups**	**Mean**	**SD**	**Mean**	**SD**	**Mean**	**SD**	**Mean**	**SD**	**Mean**	**SD**	**Mean**	**SD**
Total	-	10.0	5.7	7.3	4.9	6.2	5.3	22.8	12.3	17.1	13.3	15.6	13.2
Stage	I	9.6	5.5	6.2	3.9	4.9	4.0	21.9	16.0	15.8	12.4	13.1	10.8
	II	11.0	6.1	9.8	6.2	9.1	6.9	24.8	17.5	20.0	15.4	21.5	16.5
Pain	no	10.1	4.8	6.5	4.1	7.0	4.9	20.0	14.2	15.1	10.5	16.1	11.2
	yes	10.0	6.3	7.9	5.5	5.6	5.7	24.9	17.7	18.5	15.1	15.3	14.7
Primary disease	osteoporosis	10.7	6.7	6.8	5.5	5.6	5.0	22.8	17.0	14.5	11.3	15.1	9.2
	malignoma	9.9	5.4	7.5	4.8	6.3	5.5	22.8	16.4	17.9	13.9	15.8	14.3
Risk evaluation	low risk	11.6	7.6	7.1	6.5	6.7	6.6	27.5	21.1	17.5	16.5	19.9	17.2
	high risk	9.3	4.4	7.4	4.1	5.9	4.7	20.5	13.2	16.9	11.8	13.5	10.4
Duration of intake	short	10.3	5.2	7.7	4.4	6.2	5.2	25.6	15.7	19.7	14.0	14.9	12.2
	long	20.9	16.8	7.0	5.4	6.1	5.6	20.8	16.8	15.2	12.8	16.2	14.1
Defect size	small	10.5	5.9	6.7	4.2	5.5	3.8	23.7	15.6	16.8	11.8	15.3	11.2
	medium	9.4	5.4	7.5	5.0	6.0	6.2	19.3	14.8	14.2	12.9	13.5	12.0
	large	10.1	5.9	7.6	5.7	6.9	5.9	24.9	18.5	19.6	15.1	17.6	15.9
Need for prosthodontics	yes	11.2	5.7	8.2	5.6	6.9	6.8	26.5	16.2	18.6	13.9	18.1	16.1
	no	8.9	5.5	6.5	4.2	5.4	3.7	19.5	16.0	15.8	13.0	13.4	9.8
